# Factors related to primary cancer death and non‐primary cancer death in patients treated with stereotactic body radiotherapy for pulmonary oligometastases

**DOI:** 10.1002/cam4.3508

**Published:** 2020-10-06

**Authors:** Takaya Yamamoto, Yuzuru Niibe, Yasuo Matsumoto, Masahiko Aoki, Ryoong‐Jin Oh, Masatoki Ozaki, Mitsuru Kobayashi, Yoshihiko Manabe, Takashi Shintani, Yasuhiro Dekura, Hiroshi Onishi, Hideomi Yamashita, Keiichi Jingu

**Affiliations:** ^1^ Department of Radiation Oncology Graduate School of Medicine Tohoku University Sendai Japan; ^2^ Department of Radiology Toho University Omori Medical Center Tokyo Japan; ^3^ Department of Public Health Kurume University School of Medicine Kurume Japan; ^4^ Department of Radiation Oncology Niigata Cancer Center Niigata Japan; ^5^ Department of Radiation Oncology Hirosaki University Hirosaki Japan; ^6^ Department of Radiology Miyakojima IGRT Clinic Osaka Japan; ^7^ Department of Radiation Oncology Shizuoka City Shimizu Hospital Shizuoka Japan; ^8^ Department of Radiation Oncology Fukuyama City Hospital Fukuyama Japan; ^9^ Department of Radiology Nagoya City University Nagoya Japan; ^10^ Department of Radiation Oncology and Image‐Applied Therapy Graduate School of Medicine Kyoto University Kyoto Japan; ^11^ Department of Radiation Oncology Keiyu‐kai Sapporo Hospital Sapporo Japan; ^12^ Department of Radiology Yamanashi University Chuo Japan; ^13^ Department of Radiology University of Tokyo Tokyo Japan

**Keywords:** cancer‐specific death, non‐primary cancer death, primary cancer death, pulmonary oligometastases, stereotactic body radiotherapy

## Abstract

Cancer‐specific death (CSD) and non‐cancer‐specific death (non‐CSD) after stereotactic body radiotherapy (SBRT) for pulmonary oligometastases have not been studied in detail. The aim of this study was to determine the cumulative incidences of CSD and non‐CSD and to reveal prognostic factors. Data from a large survey of SBRT for pulmonary oligometastases were used for analyses, and patients with unknown cause of death were excluded from current analyses. CSD was primary cancer death and non‐CSD was non‐primary cancer death including a series of cancer treatment‐related deaths. Cumulative incidences were calculated using the Kaplan‐Meier method and a stratified Cox regression model was used for multivariate analyses (MVA). Fifty‐two patients with an unknown death were excluded and a total of 1326 patients was selected. CSD and non‐CSD occurred in 375 and 109 patients, respectively. The median OS period was 53.2 months and the cumulative incidences of 1‐, 3‐, and 5‐year CSD vs. non‐CSD rates were 6.5% vs. 2.3%, 29.5% vs. 8.6%, and 41.2% vs. 11.0%, respectively. In MVA, the incidence of CSD was related to performance status (1 vs. 0; *p* < 0.001, 2–3 vs. 0; *p* = 0.011), oligometastatic state (sync‐oligometastases vs. oligo‐recurrence, *p* = 0.026) and maximum tumor diameter (*p* = 0.009), and the incidence of non‐CSD was related to age (*p* = 0.001), sex (*p* = 0.030), performance status (2–3 vs. 0; *p* = 0.002), and irradiated tumor‐located lung lobe (left lower lobe vs. other lobes, *p* = 0.036). CSD was main cause of death, but non‐CSD was not rare after SBRT. Prognostic factors for CSD and non‐CSD were different, and an understanding of the factors would help in treatment.

## Graphical abstract

The incidences cancer‐specific death and non‐cancer‐specific death after stereotactic body radiotherapy for pulmonary oligometastases were 29.5 and 8.6% at 3 years, respectively. Cancer‐specific death significantly related to performance status, oligometastatic state or disease‐free interval and maximum tumor diameter, on the other hand, non‐cancer‐specific death significantly related to age and tumor‐located lung lobe.

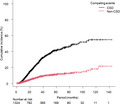

## 1 INTRODUCTION

Metastasectomy has sometimes been used for removing pulmonary metastases in cases in which the metastases are resectable and limited.^1^ There have been many reports on pulmonary metastasectomy and prognostic factors.^2^ In those studies, resectability, number of metastases, and disease‐free interval (DFI) were used to establish prognostic groups. Although those reports were published before 2000, the use of metastasectomy for various cancers has been increasing since 2000 despite various advances in systemic therapies.^3^ It has been predicted that the role of local therapies will become more important with improvements in systemic therapies for suppressing micrometastases or potential metastases and that local therapies will continue to be performed until systemic therapies have become powerful enough to eradicate macrometastases.^4^ Indication for local therapies has been increasing, and it is expected that the use of stereotactic body radiotherapy (SBRT), as an alternative to metastasectomy, will increase because of the increase in patients who are not candidates for surgery. Regarding the effectiveness of SBRT for pulmonary oligometastases, it has been reported that there was no difference in overall survival (OS) between patients who underwent pulmonary metastasectomy and patients who underwent SBRT.^5^ However, in clinical practice, since SBRT has mainly been selected for patients who were inoperable, it was expected that many cancer‐unrelated deaths after SBRT would have occurred.^6^ Therefore, the cumulative incidences of cancer‐specific death (CSD) and non‐cancer‐specific death (non‐CSD) should be analyzed separately. A large survey of SBRT for pulmonary oligometastases has been performed in Japan and the results for the primary endpoint of OS have been reported.^7^ CSD and non‐CSD were set as secondary endpoints of the study, and the aim of current study was to determine the cumulative incidences of CSD rate and non‐CSD rate and to reveal factors affecting for CSD and non‐CSD after SBRT for pulmonary oligometastases.

## 2 PATIENTS AND METHODS

### 2.1 Eligibility criteria and definitions of factors

The eligibility criteria for the study were reported elsewhere.^7^ The main inclusion criteria were that the number of pulmonary metastases was 1–5 and that the primary lesion and extrathoracic metastases needed to be controlled before SBRT. SBRT was performed from January 2004 to June 2015 and a biological effective dose (BED_10_) of 75 Gy or more. The following formula was used for calculation of BED_10_: BED_10_ = nd [1 + d/(α/β)], where n is the number of fractions, d is dose per fraction, and α/β ratio is applied for 10 Gy for the tumors.

In the current study, patients who died from an unknown cause whether primary cancer death or non‐primary cancer death were excluded from the whole data. CSDs were defined as primary cancer deaths and non‐CSDs were defined as non‐primary cancer deaths including comorbidities and age‐related death, secondary cancer death, SBRT toxicity‐related death, primary cancer treatment‐related deaths, and death related to further treatment toxicity after relapse. Adverse events were reported according to the National Cancer Institute Common Terminology Criteria for Adverse Events version 4.0 (CTCAE). DFI was from the day of surgery or the last day of radiotherapy. The oligometastatic state was classified into oligo‐recurrence, sync‐oligometastases, and unclassified oligometastases with DFIs of ≥6, 0, and <6 months, respectively. The irradiated tumor‐located lung lobe in which pulmonary oligometastasis treated by SBRT was located was classified into left lower lobe involvement and other lobes in which no irradiated tumor located based on conventional fractionated radiotherapy experience suggesting that incidental heart dose (especially the left ventricle) might affect non‐CSD.^8^ The methods for control of the primary disease included surgery, radiation, and others. Radiation included chemoradiation, radiation alone and particle therapy, and others included other control methods such as chemotherapy, radiofrequency ablation, and photodynamic therapy.

### 2.2 Statistical analysis

Time‐to‐event outcomes were calculated from the initial day of SBRT to the day that an event was confirmed, and cumulative incidences were calculated using the Kaplan‐Meier method. In univariate analyses (UVA), Gray's test was used to compare the cumulative incidences of variables. In multivariate analyses (MVA), variables with a *p* value less than 0.20 identified by UVA were put in a stratified Cox regression model using a stepwise selection to minimize the Akaike information criterion (AIC). Baseline hazard were supposed to be different from primary cancer types, therefore, primary sites were used for the stratification. Furthermore, to deal with competing risks and to keep independence of the time‐to‐event of interest, cumulative incidences of CSD and non‐CSD were estimated using Fine and Gray's proportional hazards model as sensitivity analyses.^9^ In Fine and Gray's model, primary sites were used as a factor. EZR version 1.52 (Saitama Medical Center, Jichi Medical University, Saitama, Japan), a modified version of R commander (R Foundation for Statistical Computing, Vienna, Austria), was used for analyses.^10^ A *p* value less than 0.05 was defined as significant.

### 2.3 Informed consent

The current study was a retrospective and multicenter study in Japan. The study was approved by the ethical committee of a senior facility (Ethics Committee of Toho University Omori Medical Center, reference number: 27–148). Informed consent was waived due to the retrospective study design. All of the participating institutions were guaranteed the chance to opt out of participation in this study by giving information of this study via the Internet or posters to them, and opt out consent was obtained from all patients.

## 3 RESULTS

### 3.1 Treatment outcomes

**FIGURE 1:**
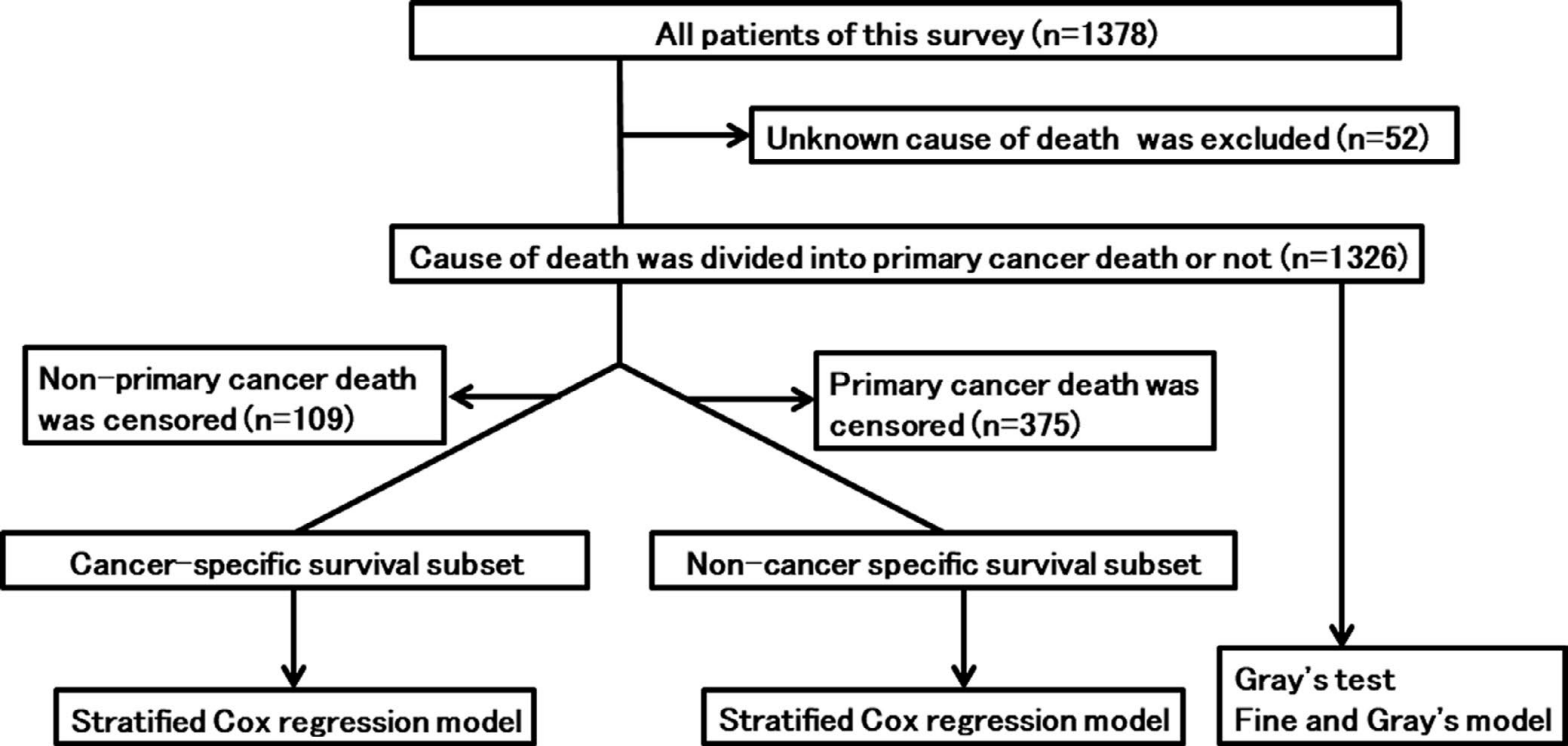
A flowchart of the identification and analyses process

**FIGURE 2:**
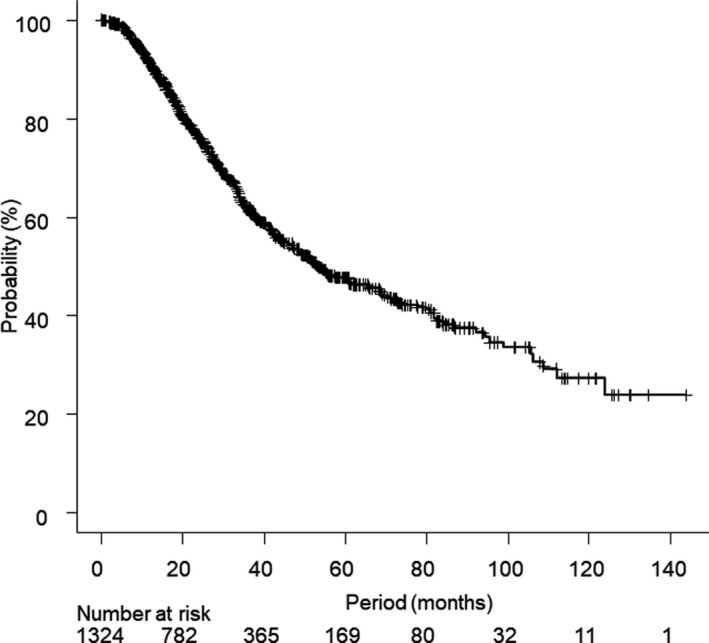
Overall survival of the current cohort

**FIGURE 3:**
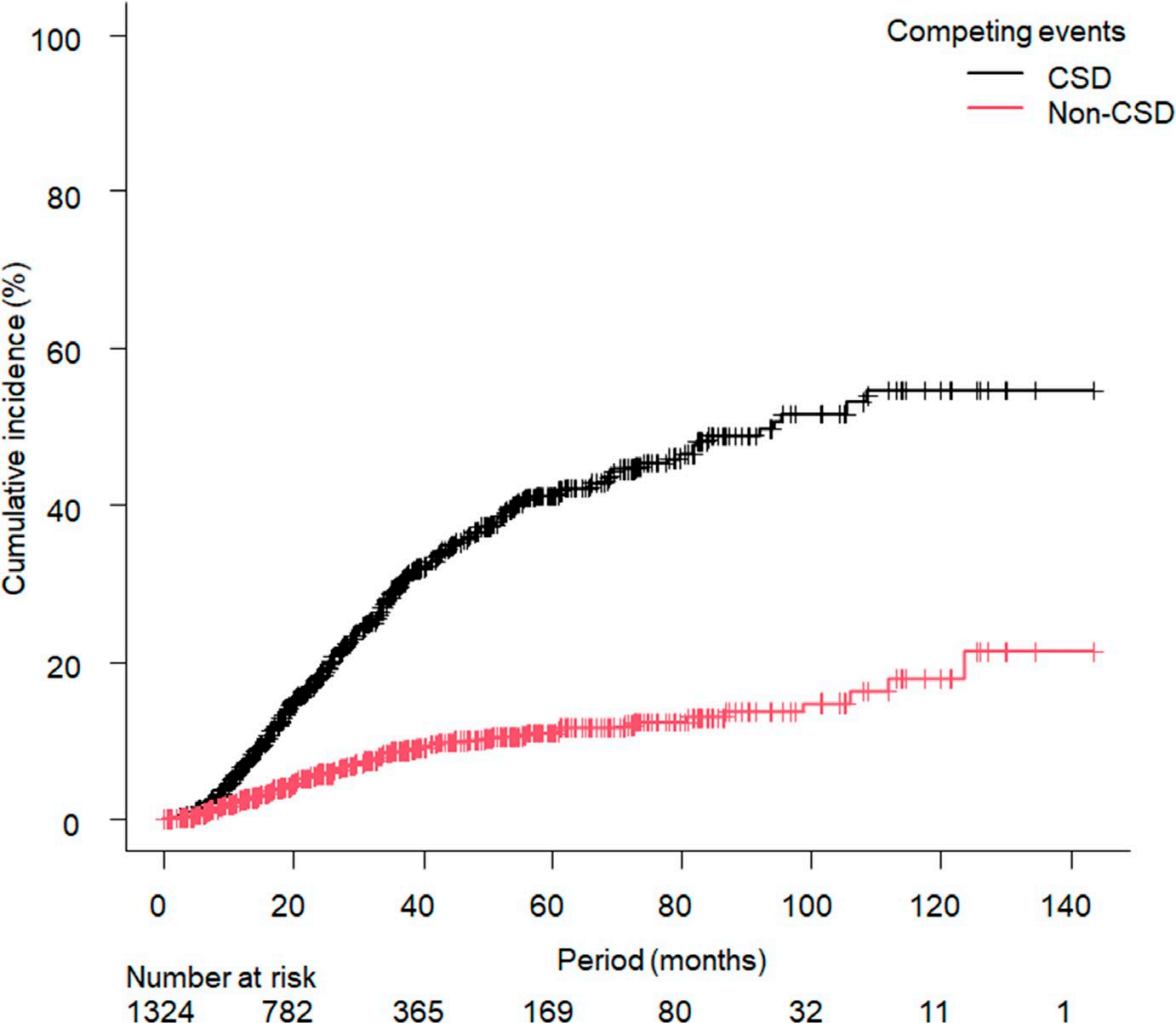
Cumulative incidences of cancer‐specific death (CSD) and non‐CSD

In the cohort of 1378 patients, 536 deceased patients were identified in the survey. Patients with an unknown cause of death (n = 52) were excluded and a total of 1326 patients were included in the current analyses (Figure 1). Characteristics of patients and pulmonary oligometastatic tumors of the current study are summarized in Table 1. Calculated BED_10_ ranged from 75.0 to 289.5 Gy, actual prescribed dose ranged 34 to 75 Gy, and number of fractions ranged from 2 to 16. During a median follow‐up period of 24.6 months (range, 0.1–143.6 months), CSD occurred in 375 patients and non‐CSD occurred in 109 patients including 10 patients with grade 5 adverse events of SBRT. The 1‐, 3‐, and 5‐year OS rates were 91.1% (95% confidence interval [CI], 89.4–92.6%), 61.9% (95% CI, 58.7–65.0%), and 47.8% (95% CI, 44.0–51.5%), respectively, and the median OS period was 53.2 months (95% CI, 47.9–65.6 months, Figure 2). The cumulative incidences of 1‐, 3‐, 5‐year CSD vs. non‐CSD rates were 6.5% (95% CI, 5.2–8.0%) vs. 2.3% (95% CI, 1.6–3.3%), 29.5% (95% CI, 26.5–32.4%) vs. 8.6% (95% CI, 6.9–10.5%), and 41.2% (95% CI, 37.6–44.8%) vs. 11.0% (95% CI, 9.0–13.3%), respectively (Figure 3).

**TABLE 1:** Characteristics of patients and pulmonary oligometastatic tumors

Characteristics	
Age, years	
Median (range)	72 (16–93)
Sex, n (%)	
Male	848 (63.9)
Female	478 (36.0)
ECOG PS, n (%)	
0	720 (56.6)
1	453 (35.6)
2–3	97 (7.6)
Primary site, n (%)	
Lung	406 (30.6)
Colorectal	336 (25.3)
Head and Neck	111 (8.3)
Esophagus	109 (8.2)
Others	364 (27.4)
Pathology, n (%)	
Squamous cell carcinoma	346 (27.4)
Adenocarcinoma	738 (58.5)
Others	176 (13.9)
Control of primary disease	
Surgery	1036 (82.8)
(Chemo)radiation	179 (14.3)
Others	36 (2.8)
Staging of primary cancer, n (%)	
cStage 1 vs. pStage 1	186 (29.4) vs. 221 (36.0)
cStage 2 vs. pStage 2	129 (20.4) vs. 133 (21.6)
cStage 3 vs. pStage 3	160 (25.3) vs. 169 (27.5)
cStage 4 vs. pStage 4	157 (24.8) vs. 90 (14.6)
Disease‐free interval, months	
Median (range)	17.9 (0–424.0)
Oligometastatic state, n (%)	
Oligo‐recurrence	985 (80.9)
Sync‐oligometastases	115 (9.4)
Unclassified oligometastases	117 (9.6)
History of local therapy prior to SBRT, n (%)	
Yes	342 (32.2)
No	720 (67.7)
Date of SBRT for initial tumor, n (%)	
2005–2009	451 (34.0)
2010–2015	875 (65.9)
Institute in which SBRT was performed, n (%)	
Academic	751 (56.6)
Nonacademic	575 (43.4)
Chemotherapy before SBRT, n (%)	
Yes	485 (36.8)
No	831 (63.1)
Chemotherapy concurrent with SBRT, n (%)	
Yes	29 (2.1)
No	1297 (97.8)
Chemotherapy after SBRT, n (%)	
Yes	189 (17.7)
No	873 (82.2)
Number of oligometastases, n (%)	
1	976 (74.0)
2	263 (19.9)
3–5	79 (5.9)
Maximum tumor diameter, cm	
Median (range)	1.5 (0.3–6.5)
SBRT dose at isocenter (BED_10_), Gy	
Median (range)	105.6 (75.0–289.5)
Irradiated tumor‐located lung lobe, tumor number (%)	
Left lower lobe involvement	224 (18.4)
Other lobes	988 (81.5)
Field coplanarity, tumor number (%)	
Coplanar field	358 (24.0)
Noncoplanar field	1131 (75.9)
Beams, tumor number (%)	
Static beam	1134 (76.0)
Arc beam	358 (23.9)

Abbreviations: BED, biological effective dose; ECOG, Eastern Cooperative Oncology Group; PS, performance status; SBRT, stereotactic body radiotherapy.

### 3.2 Univariate analyses and multivariate analyses

In UVA for CSD, age (*p* = 0.006), performance status by the Eastern Cooperative Oncology Group (ECOG PS, *p* = 0.015), primary disease site (*p* < 0.001), methods for control of primary disease (*p* < 0.001), primary disease pathology (*p* = 0.001), DFI (*p* < 0.001), oligometastatic state (*p* < 0.001), chemotherapy before and after SBRT (*p* = 0.047 and *p* < 0.001, respectively), and maximum tumor diameter (*p* < 0.001) showed significant differences (Table 2). In UVA for non‐CSD, age (*p* < 0.001), sex (*p* < 0.001), ECOG PS (*p* < 0.001), pathology (*p* = 0.008), history of local therapy for metastasis prior to SBRT (*p* = 0.019), and irradiated tumor‐located lung lobe (*p* = 0.005) showed significant differences (Table 2).

**TABLE 2:** Gray's test for cumulative incidences of variables

Variables	3‐year CSD	*p* value	3‐year non‐CSD	*p* value
Age, years				
<72	31.5		5.4	
≥72	27.3	0.006	12.0	<0.001
Sex				
Male	32.2		10.5	
Female	24.5	0.220	5.3	<0.001
ECOG PS				
0	24.9		7.5	
1	36.5		8.0	
2–3	33.8	0.015	21.6	<0.001
Primary disease site				
Lung	24.1		9.7	
Colorectal	28.9		7.5	
Head and Neck	26.9		11.8	
Esophagus	52.5		10.5	
Others	30.5	<0.001	6.8	0.197
Control of primary disease				
Surgery	27.0		6.3	
(Chemo)radiation	32.9		12.1	
Others	56.0	<0.001	8.2	0.087
Pathology				
Squamous cell carcinoma	35.5		11.9	
Adenocarcinoma	26.0		7.0	
Others	32.2	0.001	6.4	0.008
Disease‐free interval, months				
<18	35.0		8.7	
≥18	22.6	<0.001	8.7	0.685
Oligometastatic state				
Oligo‐recurrence	25.6		9.0	
Sync‐oligometastases	44.9		6.9	
Unclassified oligometastases	39.5	<0.001	7.8	0.957
History of local therapy prior to SBRT				
Yes	32.8		4.7	
No	28.8	0.147	11.0	0.019
Date of SBRT for initial tumor				
2005–2009	30.4		9.1	
2010–2015	28.8	0.379	8.2	0.635
Institute in which SBRT was performed				
Academic	31.8		8.1	
Nonacademic	27.6	0.201	9.0	0.701
Chemotherapy before SBRT				
Yes	32.2		7.4	
No	28.2	0.047	9.4	0.549
Chemotherapy concurrent with SBRT				
Yes	51.6		4.0	
No	28.9	0.052	8.7	0.913
Chemotherapy after SBRT				
Yes	42.0		4.7	
No	27.2	<0.001	10.0	0.055
Number of oligometastases				
1	28.4		9.0	
2–5	32.4	0.357	7.6	0.686
Maximum tumor diameter, cm				
<1.5	20.8		7.3	
≥1.5	36.1	<0.001	9.6	0.169
SBRT dose at isocenter (BED_10_), Gy				
<105.6	29.2		8.6	
≥105.6	28.7	0.679	8.5	0.944
Irradiated tumor‐located lung lobe				
Left lower lobe involvement	27.3		12.9	
Other lobes	30.4	0.318	8.1	0.005
Field coplanarity				
Coplanar field	27.6		9.6	
Noncoplanar field	30.1	0.255	8.4	0.549
Beams				
Static beam	29.7		9.2	
Arc beam	28.8	0.916	6.7	0.201

Abbreviations: BED, biological effective dose; ECOG, Eastern Cooperative Oncology Group; PS, performance status; SBRT, stereotactic body radiotherapy.

**FIGURE 4:**
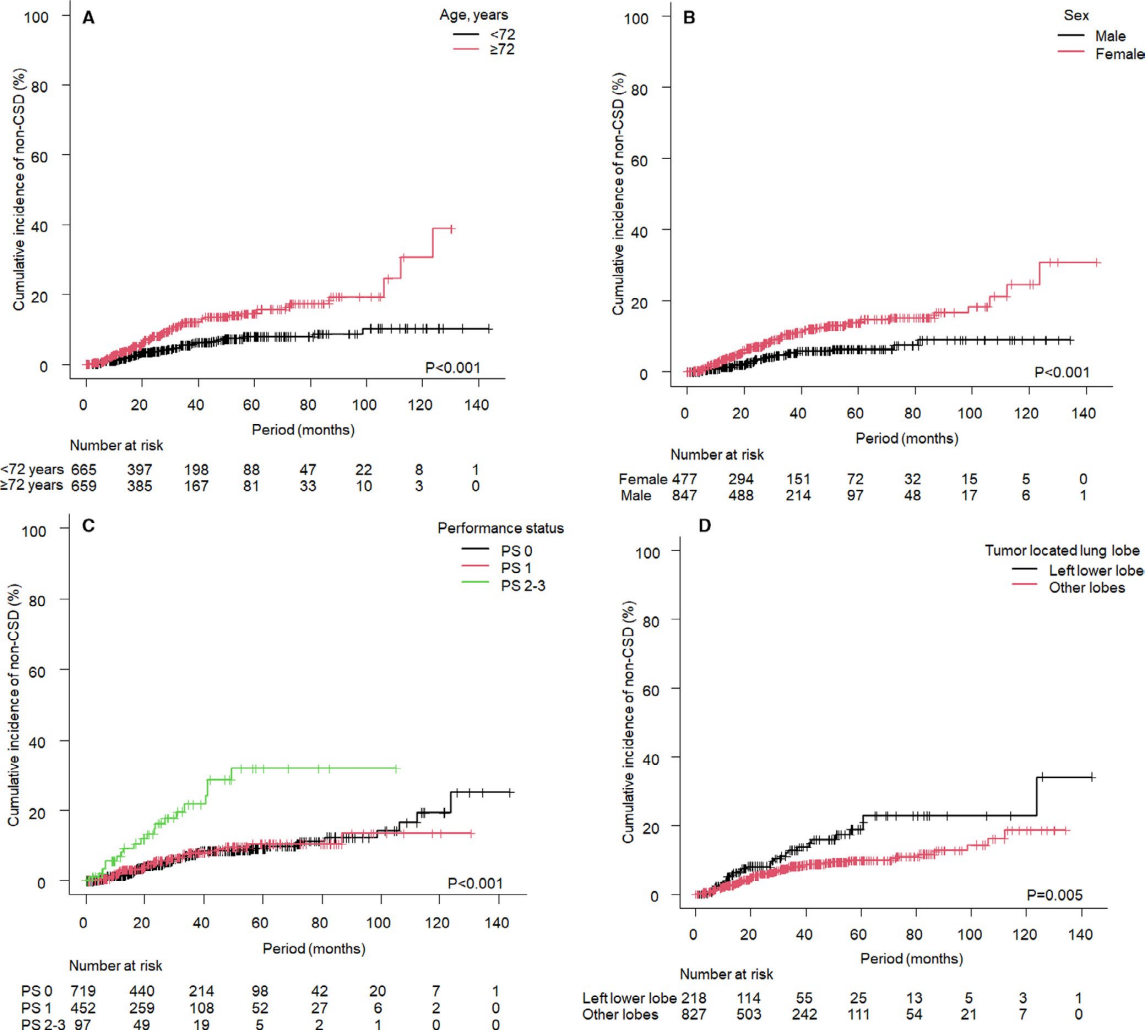
Cumulative incidences of non‐cancer‐specific death (non‐CSD) according to age, sex, performance status, and lung lobe

The results of MVA using Cox regression with stratification by primary sites are summarized in Table 3. Factors significantly related to the incidence of CSD were ECOG PS (PS 1 vs. PS 0, hazard ratio [HR]: 1.625, 95% CI: 1.252–2.108, *p* < 0.001; PS 2–3 vs. PS 0, HR: 1.889, 95% CI: 1.157–3.086, *p* = 0.001), oligometastatic state (sync‐oligometastases vs. oligo‐recurrence, HR: 1.598, 95% CI: 1.055–2.421, *p* = 0.026), and maximum tumor diameter (≥1.5 cm vs. <1.5 cm, HR: 1.405, 95% CI: 1.088–1.814, *p* = 0.009). On the contrary, the incidence of non‐CSD had significant relationships with age (≥72 years vs. <72 years, HR: 2.365, 95% CI: 1.393–4.014, *p* = 0.001), sex (male vs. female, HR: 1.943, 95% CI: 1.064–3.547, *p* = 0.030), ECOG PS (PS 2–3 vs. PS 0, HR: 2.851, 95% CI: 1.439–5.652, *p* = 0.002), and irradiated tumor‐located lung lobe (other lobes vs. left lower lobe involvement, HR: 0.577, 95% CI: 0.344–0.967, *p* = 0.036). Cumulative incidences of non‐CSD according to age, sex, PS, and lung lobe are shown in Figure 4. The results of sensitive analyses using competing‐risks regression with inclusion of primary site as a factor showed that DFI (*p* = 0.033), maximum tumor diameter (*p* = 0.049), and PS (*p* = 0.003) were significantly related to CSD, and age (*p* = 0.001), pathology (*p* < 0.001), and irradiated tumor‐located lung lobe (*p* = 0.039) were significantly related to non‐CSD.

**TABLE 3:** Results of multivariate stratified Cox regression analyses

Variables	Cancer‐specific death		Non‐cancer‐specific death	
	HR (95% CI)	*p* value	HR (95% CI)	*p* value
Age, years				
≥72 vs. <72	Not selected		2.365 (1.393–4.014)	0.001
Sex				
Male vs. female	Not selected		1.943 (1.064–3.547)	0.030
ECOG PS				
1 vs. 0	1.625 (1.252–2.108)	<0.001	1.047 (0.619–1.768)	0.864
2–3 vs. 0	1.889 (1.157–3.086)	0.011	2.851 (1.439–5.652)	0.002
Disease‐free interval, months				
≥18 vs. <18	0.778 (0.579–1.046)	0.096	Not selected	
Oligometastatic state				
Sync‐oligo vs. oligo‐rec	1.598 (1.055–2.421)	0.026	Not selected	
Unclassified vs. oligo‐rec	1.365 (0.956–1.812)	0.126		
Chemotherapy after SBRT				
Yes vs. No	1.316 (0.956–1.812)	0.091	Not selected	
Maximum tumor diameter, cm				
≥1.5 vs. <1.5	1.405 (1.088–1.814)	0.009	Not selected	
Irradiated tumor‐located lung lobe				
Other lobes vs. left lower lobe involvement	Not selected		0.577 (0.344–0.967)	0.036

Abbreviations: ECOG, Eastern Cooperative Oncology Group; oligo‐rec, oligo‐recurrence; PS, performance status; SBRT, stereotactic body radiotherapy; sync‐oligo, sync‐oligometastases.

## 4 DISCUSSION

Metastasis‐directed therapy has progressed with surgical experience, with widespread recognition of the concept of oligometastasis and with technological developments in radiotherapy.^1^, ^2^, ^4^, ^11^ Since SBRT has been used worldwide, many results of SBRT as a metastasis‐directed therapy have been reported.^6^, ^12^, ^13^, ^14^ Because SBRT has been mainly performed for patients who were not candidates for surgery, the concern for non‐CSD generated the hypothesis of the current study. As a result, the majority of the patients died from primary cancer, therefore, treatment for primary cancer should remain a high priority. But, the incidence of non‐CSD was not rare and this large survey enabled analysis of non‐CSD and provided interesting and informative results. To the best of our knowledge, factors affecting non‐CSD after SBRT for pulmonary oligometastases were first revealed.

MVA for non‐CSD revealed that aged patients (≥72 years old), male sex, poor performance status (PS 2–3), and left lower lobe involvement of the irradiated tumor were related to a high incidence of non‐CSD. Age and left lower lobe involvement were confirmed by sensitivity analyses. The results for age were naturally expected from life expectancy and relatively lower tolerance of elder patients for invasiveness.

The left lower lobe involvement was analyzed to determine the effect of incidental heart dose on non‐CSD as mentioned in Section 2. There has recently been accumulating evidence of cardiac toxicity after radiotherapy for locally advanced non‐small‐cell lung cancer, and cardiac dose and tumor location in left lower lobe have been shown to be important factors.^8^, ^15^, ^16^, ^17^ The effect of the lobe in which the tumor was located (left lower lobe or other lobes) on non‐CSD was, therefore, investigated partly in substitution for cardiac dose assessment which was not investigated in the survey. It was found that location of the irradiated tumor in the left lower lobe significantly increased the risk of non‐CSD. Cardiac dose‐volume analyses of SBRT have not shown a relationship with cardiac events or overall survival.^18^ But, in conventional fractionated radiotherapy, it was reported that maximum dose to the left atrium and the dose to 90% of the superior vena cava were associated with non‐cancer death.^19^ Cardiac dose assessment would be more sensitive to cardiotoxicity than tumor location (left lower lobe vs. other lobes).^20^ As patients live longer due to the progress in radiotherapy and systemic therapy, cardiac doses might become more important. To determine another incidental radiation effect, primary disease controlled by radiation or not and SBRT technique (field coplanarity and static or arc beam) were also investigated, but they showed no significance.

The 3‐year cumulative incidence of non‐CSD according to pathology were 11.9% in squamous cell carcinoma, 7.0% in adenocarcinoma, and 6.4% in other pathology (*p* = 0.008), but pathology showed significance only in sensitivity analyses. This is why Cox regression was stratified by primary sites and the concept of field cancerization would be accountable for the reason.^21^ Large areas of the head and neck mucosa are affected by exposure to many carcinogens, leading to the development of a precancerous lesion that changes into malignancy. Actually, it has been reported that second primary malignancy was the leading cause of non‐primary cancer death in patients with head and neck squamous cell cancers who survived for 3 years or longer.^22^ Carcinogens in cigarette and alcohol would play such a role in the upper respiratory and upper digestive tracts.^23^, ^24^, ^25^ Without stratification by primary sites, it is thought that squamous cell carcinoma emerged as a representative prognostic factor since histories of smoking and alcohol drinking were not investigated in the survey.

The results of this study revealed that ECOG PS 0 and smaller maximum tumor diameter were significantly associated with a lower incidence of CSD. Oligo‐recurrence showed significance in stratified Cox regression and longer DFI (≥18 months) showed significance in competing risk regression, which was thought to be valid because all the case with DFI of 6 months or longer were classified into oligo‐recurrence. The results for ECOG PS, oligometastatic state, DFI, and maximum tumor diameter confirmed that previous findings were also applied to CSD.^6^, ^7^, ^26^, ^27^, ^28^ ECOG PS has often been a problem in cancer treatments.^29^ The invasiveness of SBRT is possibly a burden even for patients with poor PS as well as systemic therapy would be a burden for patients with poor PS.^30^, ^31^ Poor PS might also reflect to some extent the effect of the patient's comorbidities, which were not investigated in the survey.

There were several limitations in the current study. There was uncontrollable confounding by indications because of the retrospective nature of the study. Unknown cause of death was excluded from analyses, therefore, survival outcomes were overestimated. Comorbidities, smoking and alcohol habits, peripheral or central tumor location, chemotherapy regimen, SBRT dose‐volume data, some prior radiotherapy dose‐volume data, and cause of non‐CSD were not investigated in the survey. Some short‐term follow‐up data were included and various treatment protocols at the institutions were included. Statistically, further analyses using the stratified Fine and Gray's model are desirable.

## 5 CONCLUSIONS

In conclusion, the results showed that main cause of death after SBRT for pulmonary oligometastases was primary cancer death, and the 3‐year CSD and non‐CSD rates were 29.5 and 8.6%, respectively. Treatment for primary cancer should remain a high priority, but non‐CSD was not rare. Factors significantly related to the incidence of CSD are PS, oligometastatic state or DFI and maximum tumor diameter, and these results are reasonable considering previous findings. On the contrary, factors significantly related to the incidence of non‐CSD were age and irradiated tumor‐located lung lobe, and these results are interesting and informative. Dose constraints for the heart might contribute to a reduction in the risk of non‐CSD in patients with tumor location in the left lower lobe.

## CONFLICT OF INTERESTS

YN has received lecturer fees from Janssen Pharmaceutical K.K. TY, YM, MA, RO, MO, MK, YM, TS, YD, HO, HY, and KJ have nothing to disclose.

## Data Availability

Due to the nature of this research, participants of this study did not agree for their data to be shared publicly. Therefore, the data contained in the claims database cannot be made publicly available at this time.
